# Diagnosis of Pneumoperitoneum Using POCUS

**DOI:** 10.24908/pocusj.v11i01.19753

**Published:** 2026-04-22

**Authors:** Maria Paulina Maya Jaramillo, Alejandro Cardozo Ocampo

**Affiliations:** 1 Medical student, EIA University in Medellín, Colombia; 2 Emergency Physician, Neurological Institute of Colombia

**Keywords:** Pneumoperitoneum, Point of care ultrasound, POCUS, Enhanced peritoneal stripe sign

## Abstract

Point of care ultrasound (POCUS) of the nontraumatic acute abdomen is limited by its low sensitivity for detecting pneumoperitoneum. However, POCUS signs that suggest the presence of free intraperitoneal air have been described in the literature, such as the enhanced peritoneal stripe. We describe a patient who arrived at our emergency department with an acute abdomen that had been present for several hours. POCUS suggested pneumoperitoneum, which was confirmed by abdominal X-ray and the operative finding of a perforated peptic ulcer. This case highlights the potential role of POCUS as a rapid, non-invasive bedside diagnostic tool in emergency settings, especially when access to other diagnostic aids may be limited or delayed. Recognizing characteristic POCUS signs, including an enhanced peritoneal stripe and reverberation artifacts, can guide early decision-making and expedite surgical intervention. Timely identification of pneumoperitoneum is crucial, because delays in diagnosis are associated with increased morbidity and mortality. This case report reinforces the value of integrating POCUS into the initial evaluation of acute abdominal pain, suggesting its use as an adjunct to traditional imaging methods in the emergency department.

## Introduction

The initial evaluation of patients with nontraumatic acute abdomen in the emergency department can present a diagnostic challenge. This is particularly true when an immediate surgical condition such as visceral perforation is suspected, which manifests as free intraperitoneal air (pneumoperitoneum) on imaging. Traditionally, this diagnosis relies on abdominal radiography or computed tomography (CT); however, the difficulty of interpreting supine radiographs and the limited availability of CT scanners in some emergency departments enhance the value of point of care ultrasound (POCUS) as an initial diagnostic tool.

POCUS has been shown to improve bedside diagnostic accuracy, reduce the need for additional imaging, and expedite clinical decision making [1]. In recent years, its application for identifying pneumoperitoneum has been increasingly described in case series and literature reviews. The main sonographic signs include the enhanced peritoneal stripe sign, A-line–type reverberation artifacts along the hepatic margin, and the gut point—the abdominal analogue of the pulmonary “lung point.” Technical maneuvers such as left lateral decubitus positioning, the “scissors maneuver,” and the “lung curtain sign” can improve the visualization of free intraperitoneal air [2,3].

Here, we report the case of a patient presenting with an acute abdomen in whom POCUS was the first diagnostic tool suggesting pneumoperitoneum and the need for urgent surgery. This case provides four distinctive contributions: (1) early POCUS detection of free air in the context of a perforated peptic ulcer during initial evaluation (POCUS as the first guiding study before radiography or CT), (2) the practical use of the lung curtain sign and dynamic compression maneuvers to differentiate between free gas and intraluminal gas, (3) to our knowledge, the first reported case in Colombia of pneumoperitoneum primarily diagnosed by POCUS, and (4) proposes a practical and reproducible ultrasound approach aimed at improving the consistency of examinations in emergency settings.

## Case presentation

A 68-year-old man with a history of high blood pressure and occasional smoking presented to the emergency department. He had sudden onset of epigastric abdominal pain that intensified, reaching a pain score of 10/10 on a pain scale. This pain was associated with nausea without vomiting or other gastrointestinal symptoms. Physical examination revealed a heart rate of 96 bpm, blood pressure of 149/72 mm Hg, afebrile, and no requirement for supplemental oxygen. Physical examination revealed diffuse abdominal pain with guarding and rebound, especially in the upper region.

**Figure 1.**
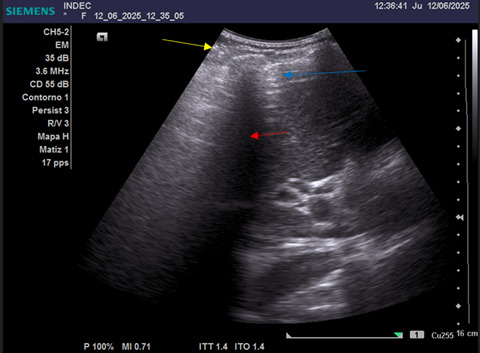
Abdominal point of care ultrasound (POCUS) with patient in the supine position. A low-frequency convex transducer (3.5–3.6 MHz) was used in the abdominal preset according to emergency POCUS protocol. The probe was placed in the right subcostal region with the marker oriented cephalad (longitudinal view). The image shows: yellow arrow = pleural line; red arrow = acoustic rib shadow; blue arrow = liver edge with A-line–type reverberation artifacts suggestive of free intraperitoneal air.

**Figure 2.**
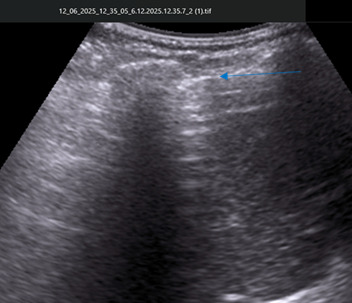
Abdominal point of care ultrasound (POCUS) image, magnified region of interest from Figure 1, same patient and same acoustic window. The blue arrow highlights the enhanced peritoneal stripe and reverberation artifacts along the hepatic margin, consistent with pneumoperitoneum. Supine position; convex transducer 3.6 MHz; longitudinal orientation.

**Figure 3.**
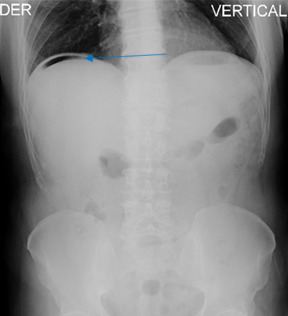
X-ray subdiagrammatic air (arrow).

Due to clinical suspicion of a surgical emergency, Focused Assessment with Sonography for Trauma (FAST) examination was performed and negative for free fluid. However, in an epigastric and right upper quadrant view, POCUS showed the liver topography, which revealed images like the pulmonary A-line pattern, suggestive of air (Figure 1). In order to differentiate the lung from the liver, the “curtain sign” was verified and the POCUS image was enlarged. This suggested abdominal air not in the thorax (Figure 2). The standing abdominal X-ray (Figure 3) confirmed the POCUS finding so the patient was taken to laparotomy. A perforated peptic ulcer was found; after a five-day period of hospitalization, the patient was discharged without complications.

## Discussion

The timely diagnosis of pneumoperitoneum in patients with clinical signs of peritoneal irritation remains a challenge in emergency departments, especially when imaging resources are limited and rapid and accurate evaluation is required. In this context, POCUS has emerged as an accessible, noninvasive, and effective tool for the initial assessment of the acute abdomen [4–7].

In the assessment of possible pneumoperitoneum, the scan can be performed with a convex or phased array transducer, with greater sensitivity in the epigastric and subcostal windows, especially on the right [7–9]. Previous studies of these cases have described POCUS findings of free intraperitoneal air and an A-line pattern similar to that seen in thoracic ultrasonographic semiology, known as the enhanced peritoneal stripe sign [8]. As in our case, this is like a pulmonary A-line pattern but in the peritoneum at its hepatic or splenic border. In this same location, other authors have suggested an artifact reverberation type with a ring down like comet tails that begin in the peritoneum [7]. These findings, like those found in pulmonary evaluation with POCUS, reinforce the value of POCUS as a tool in the early detection of pneumoperitoneum in patients with acute abdomen. Reported sensitivity and specificity are 95% and 81%, respectively [7]. This supports using POCUS as an initial screening tool for pneumoperitoneum as a rapid, non-invasive, and bedside evaluation.

**Figure 4.**
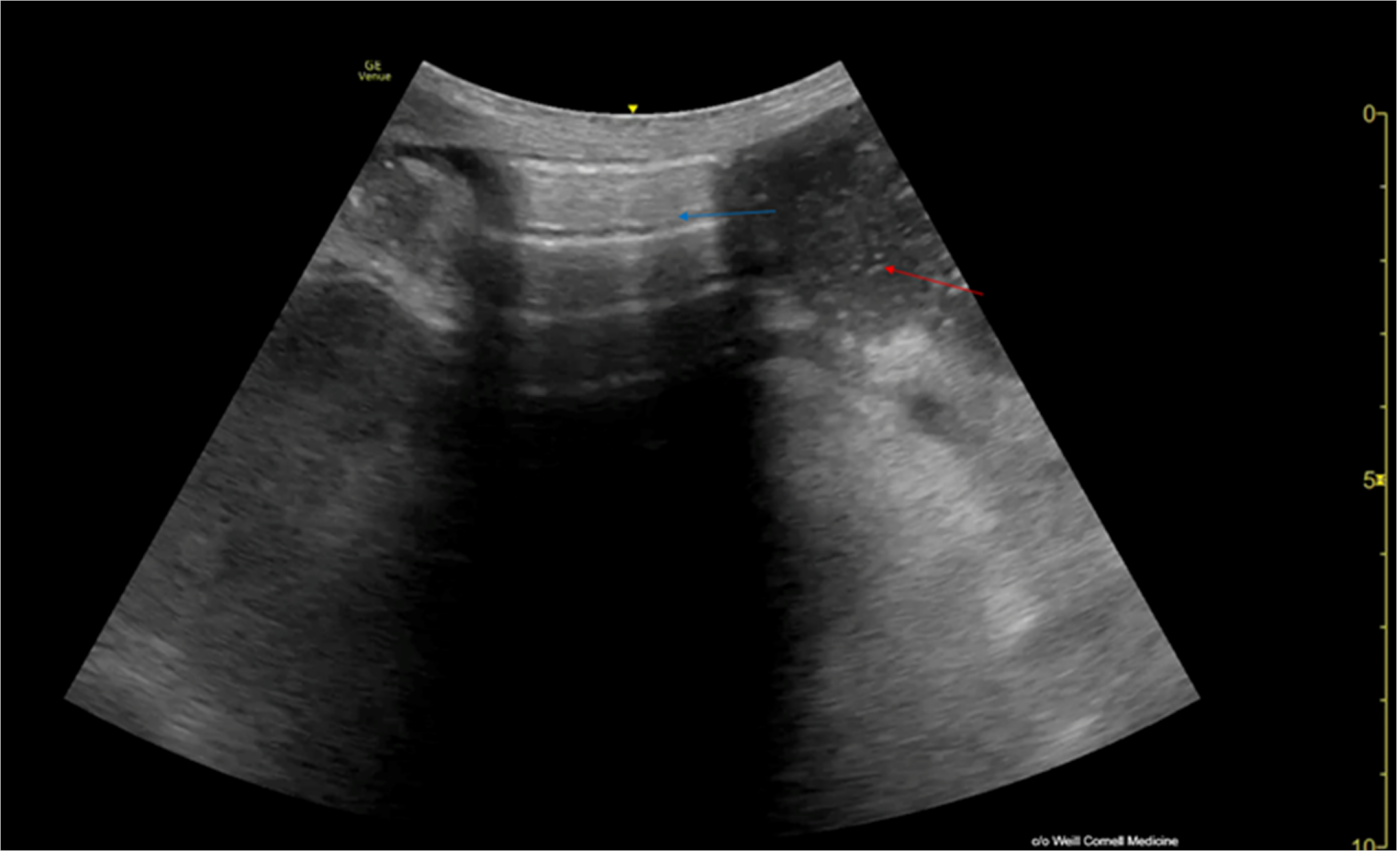
Abdominal point of care ultrasound (POCUS) with patient in the supine position. A convex transducer in transverse orientation in the abdominal preset (2.0 MHz) is used. The image suggests dilated bowel loops compatible with intestinal obstruction; the red arrow indicates a fluid and gas-filled dilated loop. The blue arrow indicates air in the bowl. The presence of A-lines mimics pneumoperitoneum. The image is courtesy of Tanping Wong, MD of Weill Cornell Medicine.

A key use of POCUS is differentiating the artifacts associated with pneumoperitoneum from dilated bowel loops in intestinal obstruction (Figure 4). On POCUS, these appear as fluid-filled loops, wall thickening, and decreased or absent peristalsis. Whereas in pneumoperitoneum, A-line pattern artifacts are located superficially near the liver, and shift with pressure exerted by a transducer.

Moriwaki et al. provided essential criteria for avoiding a false positive interpretation of pneumoperitoneum. Intraluminal air never overlaps the ventral surface of the liver, unlike free air that collects just below the hepatic peritoneum. Furthermore, air within the gastrointestinal tract maintains continuity with the loops and does not change or shift with transducer compression, whereas free air exhibits mobility and its POCUS pattern can be modified by applied pressure. This distinction helps to identify findings that can mimic pneumoperitoneum and to improve the diagnostic accuracy of POCUS when evaluating the acute abdomen.

Finally, it should be noted that like other POCUS applications in gastroenterology, there are still no standardized protocols or universal training in abdominal POCUS for the detection of pneumoperitoneum [8]. However, we suggest the following approach for detecting pneumoperitoneum during POCUS: with the patient in the supine position and the legs bent to promote abdominal muscle relaxation, begin the examination with a low-frequency convex probe (3.5 MHz). Place it longitudinally in the epigastrium and then slide it toward the right subcostal margin to identify the enhanced peritoneal stripe sign or A-lines as reverberation artifacts along the hepatic margin. Additionally, we recommend performing the scissors maneuver to differentiate air-filled intestinal loops from free intraperitoneal air. In the scissor maneuver, the patient is placed on the left lateral position, thus allowing the peritoneal free air to levitate to the right upper quadrant of the abdomen. A-line pattern reverberation artifacts can then be seen overlying the liver. The disappearance of these A-lines with gentle pressure and their reappearance with the release of pressure indicate the presence of mobile free air within the peritoneal cavity.

Furthermore, structured training in the recognition of specific POCUS signs—such as the enhanced peritoneal stripe—and the use of the lung curtain sign to differentiate the lung from the liver can improve diagnostic accuracy and promote broader adoption of this technique.

In the clinical case presented above, an enhanced peritoneal stripe sign like the pulmonary A-line pattern was identified at the hepatic edge, suggestive of pneumoperitoneum [1,4,7].

These findings reinforce the value of POCUS in the assessment of non-traumatic acute abdomen in the semiology that suggests pneumoperitoneum.

## References

[1] Mateer J, Plummer D, Heller M, Olson D, Jehle D, Overton D, Gussow L. Model curriculum for physician training in emergency ultrasonography. Ann Emerg Med. 1994;23(1):95–102. doi:10.1016/s0196-0644(94)70014-1 8273966

[2] Bacci M, Salas M, Perera P. Pneumoperitoneum detected by point-of -care ultrasound: a case report. Cureus. 2020;12(3):e7301. doi:10.7759/cureus.7301 32313742 PMC7163341

[3] Taylor RA, Bundy A, Smith S. Detection of pneumoperitoneum using bedside ultrasound: case report and review of the literature. Ultrasound J. 2020;12(1):17. doi:10.1186/s13089-020-00195-z32246214

[4] Chao A, Gharahbaghian L, Perera P. Diagnosis of pneumoperitoneum with bedside ultrasound. West J Emerg Med. 2015;16(2):302. doi:10.5811/westjem.2014.12.24945 25834673 PMC4380382

[5] Jones R. Recognition of pneumoperitoneum using bedside ultrasound in critically ill patients presenting with acute abdominal pain. Am J Emerg Med. 2007;25(7):838–41. doi:10.1016/j.ajem.2007.02.004. PMID: 17870492. 17870492

[6] Chen SC, Wang HP, Chen WJ, Lin FY, Hsu CY, Chang KJ, Chen WJ. Selective use of ultrasonography for the detection of pneumoperitoneum. Acad Emerg Med. 2002;9(6):643–5. doi:10.1111/j.1553-2712.2002.tb02307.x 12045083

[7] Nazerian P, Tozzetti C, Vanni S, Bartolucci M, Gualtieri S, Trausi F, Vittorini M, Catini E, Cibinel GA, Grifoni S. Accuracy of abdominal ultrasound for the diagnosis of pneumoperitoneum in patients with \acute abdominal pain: a pilot study. Crit Ultrasound J. 2015;7(1):15. doi:10.1186/s13089-015-0032-6 26443344 PMC4595408

[8] Moriwaki Y, Sugiyama M, Toyoda H, Kosuge T, Arata S, Iwashita M, Tahara Y, Suzuki N. Ultrasonography for the diagnosis of intraperitoneal free air in chest–abdominal–pelvic blunt trauma and critical acute abdominal pain. Arch Surg. 2009;144(2):137–141. doi:10.1001/archsurg.2008.553 19221324

[9] Shankar N, Kuo L, Krugliak Cleveland N, Galen B, Samel NS, Perez-Sanchez A, Nathanson R, Coss E, Echavarria J, Rubin DT, Soni NJ. Point-of-Care Ultrasound in Gastroenterology and Hepatology. Clin Gastroenterol Hepatol. 2025;23(8):1277–1290. doi:10.1016/j.cgh.2024.09.040. 39793722

